# Modulation of injectable hydrogel properties for slow co‐delivery of influenza subunit vaccine components enhance the potency of humoral immunity

**DOI:** 10.1002/jbm.a.37203

**Published:** 2021-05-06

**Authors:** Olivia M. Saouaf, Gillie A. Roth, Ben S. Ou, Anton A. A. Smith, Anthony C. Yu, Emily C. Gale, Abigail K. Grosskopf, Vittoria C.T.M. Picece, Eric A. Appel

**Affiliations:** ^1^ Department of Materials Science & Engineering Stanford University Stanford California USA; ^2^ Department of Bioengineering Stanford University Stanford California USA; ^3^ Department of Biochemistry Stanford University School of Medicine Stanford California USA; ^4^ Department of Chemical Engineering Stanford University Stanford California USA; ^5^ Department of Chemistry & Applied Biosciences ETH Zürich Zürich Switzerland; ^6^ Institute for Immunity, Transplantation & Infection Stanford University School of Medicine Stanford California USA; ^7^ ChEM‐H Institute Stanford University Stanford California USA; ^8^ Department of Pediatrics – Endocrinology Stanford University School of Medicine Stanford California USA

**Keywords:** biomaterials, hydrogels, immunoengineering, infectious disease, vaccines

## Abstract

Vaccines are critical for combating infectious diseases across the globe. Influenza, for example, kills roughly 500,000 people annually worldwide, despite annual vaccination campaigns. Efficacious vaccines must elicit a robust and durable antibody response, and poor efficacy often arises from inappropriate temporal control over antigen and adjuvant presentation to the immune system. In this work, we sought to exploit the immune system's natural response to extended pathogen exposure during infection by designing an easily administered slow‐delivery influenza vaccine platform. We utilized an injectable and self‐healing polymer‐nanoparticle (PNP) hydrogel platform to prolong the co‐delivery of vaccine components to the immune system. We demonstrated that these hydrogels exhibit unique dynamic physical characteristics whereby physicochemically distinct influenza hemagglutinin antigen and a toll‐like receptor 7/8 agonist adjuvant could be co‐delivered over prolonged timeframes that were tunable through simple alteration of the gel formulation. We show a relationship between hydrogel physical properties and the resulting immune response to immunization. When administered in mice, hydrogel‐based vaccines demonstrated enhancements in the magnitude and duration of humoral immune responses compared to alum, a widely used clinical adjuvant system. We found stiffer hydrogel formulations exhibited slower release and resulted in the greatest improvements to the antibody response while also enabling significant adjuvant dose sparing. In summary, this work introduces a simple and effective vaccine delivery platform that increases the potency and durability of influenza subunit vaccines.

## Graphical abstract

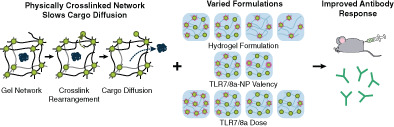

## 1 INTRODUCTION

Even our ever‐changing yearly flu vaccines are insufficient to stop the roughly 500,000 deaths occurring annually worldwide from seasonal influenza. Moreover, new influenza strains with all of the hallmarks of a pandemic threat continue to emerge, including the recently described Eurasian avian‐like H1N1 swine influenza strain dubbed G4.^1^ A successful vaccine providing potent and durable humoral immune responses against influenza must generate broadly neutralizing antibodies against the viral envelope protein hemagglutinin (HA).^2^, ^3^ High quality antibodies are generally characterized by high degrees of somatic hypermutation and commensurate affinity maturation that generally occurs in germinal centers (GCs) in the lymph nodes following immunization. Numerous efforts have been taken to engineer novel vaccination approaches to promote higher degrees of affinity maturation or to target highly conserved epitopes by engineering multivalent antigen nanoparticles^4^, ^5^ or novel antigen proteins.^6^, ^7^

In addition to antigen engineering, many efforts have focused on the development of more potent adjuvants, such as toll‐like receptor agonists (TLRa) to improve the immunogenicity of the subunit antigens. Unfortunately, while reports indicate that subunit vaccines comprising multiple TLRa molecules elicit better immune memory and stronger antibody responses,^8^, ^9^, ^10^ it was recently reported that numerous highly potent adjuvant systems increase binding antibody titer but do not affect levels of somatic hypermutation when used in HIV vaccines in non‐human primates.^11^ These results indicate that adjuvants alone, even potent TLRa adjuvants, may not be sufficient to spur high quality immune responses, highlighting that a critical need exists for improved spatiotemporal control over vaccine delivery.

Further vaccine engineering has demonstrated that time‐controlled release is favorable for eliciting stronger immune system responses.^12^ It has recently been demonstrated that sustained release of an HIV vaccine using a surgically implantable osmotic pump leads to more robust and durable GC responses, higher antibody titers, and the targeting of a more diverse set of epitopes and better virus neutralization than standard bolus administration of the same vaccine.^13^ While this work clearly highlighted the importance of sustained vaccine exposure on improving humoral immune responses, the pump system is cumbersome, and its implantation is invasive. Injectable depot technologies represent a promising, minimally‐invasive approach to prolonged vaccine delivery.^14^, ^15^ Our group previously reported the use of dynamic, injectable polymer nanoparticle (PNP) hydrogel platform for prolonged co‐delivery of subunit vaccine components to enhance humoral immune responses.^14^, ^16^ These PNP hydrogels consist of polymer strands dynamically cross‐linked by multivalent non‐covalent interactions with nanoparticles (NPs), allowing for dynamic detachment and reattachment to achieve injectability followed by robust depot formation.^17^ This work reported that physicochemically distinct subunit vaccine cargo ovalbumin and poly(I:C), a potent TLR3a, could be co‐delivered over prolonged time‐frames, enhancing the magnitude and duration of GC responses and antibody responses, leading to a 1,000‐fold increase in antibody affinity maturation.^14^

To achieve potent, durable, and high‐quality antibody responses to influenza vaccines, it appears that three parameters are necessary: (a) sustained vaccine exposure to prolong affinity maturation, (b) the use of adjuvants, and (c) precise co‐delivery of subunit vaccine components. Currently, the relationship between timeframe of vaccine exposure and the resulting immune response is poorly understood. Moreover, while numerous approaches have been shown to improve antigen delivery,^18^, ^19^, ^20^, ^21^ often the challenge of sustained co‐delivery of antigen and adjuvant persists. A platform that allows for controlled release of multiple components is necessary to elicit potent responses. Yet, controlled encapsulation and sustained release of reagents differing in chemistry and size has proven challenging.^8^ Indeed, many studies evaluating combinations of adjuvants have focused on those of similar physicochemical properties, such as CpG (TLR9a) and poly(I:C) (TLR3a) since they are both nucleic acid polymers or MPL (TLR4a) and imidazoquinolines (TLR7a or TLR7/8a) since they are both hydrophobic, and new materials technologies are required to generate opportunities to evaluate novel, synergistic adjuvant pairings^22^, ^23^ and improve subunit vaccine delivery.

When considering the use of hydrogels as delivery vehicles for subunit vaccines, it is of utmost importance to evaluate the mechanical properties of the gel. While previous works acted as a proof of concept for the use of PNP hydrogels as a vaccine delivery platform,^24^ the relationship between material properties and the resulting immune response was not evaluated. Alteration of the hydrogel formulation will, for example, affect the diffusivity of vaccine cargo, thereby changing the timeframe of vaccine exposure. Additionally, the conjugation of TLR7/8a to the PEG–PLA NPs that act as a structural motif within the hydrogel network allows this small molecule adjuvant to diffuse at the same rate as an entrapped HA antigen protein. By tuning the diffusion kinetics of these cargo, we have shown it is possible to optimize the hydrogel formulation to achieve temporal and spatial co‐delivery of adjuvant and influenza antigen.

**FIGURE 1:**
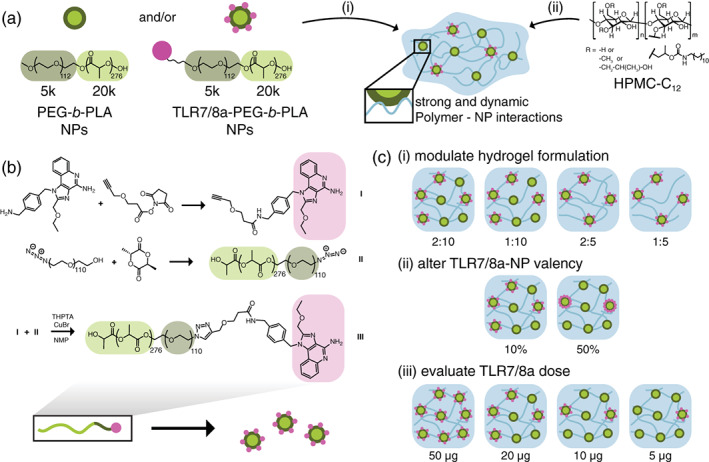
Fabrication of Polymer‐Nanoparticle (PNP) Hydrogel and TLR7/8a Nanoparticles. (a) PNP hydrogels are formed when (i) poly(ethylene glycol)‐*b*‐poly(lactic acid) (PEG–PLA) nanoparticles (NPs) or TLR7/8 agonist (TLR7/8a) conjugated PEG–PLA NPs are combined with (ii) dodecyl‐modified hydroxypropylmethylcellulose (HPMC‐C_12_). Multivalent and dynamic non‐covalent interactions between the polymer and NPs constitute physical cross‐links within the hydrogel structure. Vaccine cargo can be added to the aqueous NP solution before mixing. (b) NHS coupling of TLR7/8a to alkyne (**I**), and the coupling to azide‐terminated PEG–PLA (**II**) to make PEG–PLA with the TLR7/8a (pink) presenting on the PEG (dark green) terminus of the block copolymer (**III**). This polymer can then be mixed with standard PEG–PLA and then nanoprecipitated into water to form TLR7/8a‐NPs with a TLR7/8a valency defined by the physical mixture of the two polymers. (c) Gel formulations were varied by (i) changing the ratio of polymer: NP: solvent where 2:10 denotes a formulation comprising 2 wt% HPMC‐C_12_: 10 wt% NP: 88 wt% solvent, (ii) altering the TLR7/8a‐NP valency, and (iii) altering the total dose of entrapped TLR7/8a molecules

In this work, we have sought to engineer PNP hydrogels to improve humoral immune response to influenza vaccines comprising HA and a potent TLR7/8a adjuvant, (1‐(4‐[aminomethyl]benzyl)‐2‐(ethoxymethyl)‐1H‐imidazo[4,5‐c]quinolin‐4‐amine) (Figure 1), which is a derivative of Resiquimod (R848). We build on recent work where we demonstrated that by chemically conjugating the TLR7/8a to the NP structural motif of these hydrogels it is possible to achieve sustained antigen and adjuvant co‐delivery.^24^ We show that modulation of the PNP hydrogel formulation affects the structural and mechanical properties of the hydrogel, imparting control over cargo diffusion and achievable timescales of vaccine cargo co‐delivery. When administered in mice, hydrogel‐based vaccines demonstrate enhancements in the magnitude and duration of humoral immune responses compared to alum, a widely used clinical adjuvant system. We find stiffer hydrogel formulations exhibiting slower release yield the greatest improvements to the antibody response while also enabling significant adjuvant dose sparing. In summary, this work introduces a simple and effective vaccine delivery platform that increases the potency and durability of influenza subunit vaccines.

## 2 MATERIALS AND METHODS

### 2.1 Materials

HPMC (USP grade), N,N‐Diisopropylethylamine (Hunig's base), hexanes, diethylether, N‐methyl‐2‐pyrrolidone (NMP), dichloromethane (DCM), lactide (LA), 1‐dodecylisocynate, diazobicylcoundecene (DBU), and fluorescein isothiocyanate‐dextrans (FITC‐Dex) were purchased from Sigma Aldrich and used as received. Monomethoxy‐PEG (5 kDa) was purchased from Sigma Aldrich and was purified by azeotropic distillation with toluene prior to use. AF647‐DBCO was purchased from Thermo Fisher and used as received. HIS‐Lite‐Cy3 Bis NTA‐Ni Complex was purchased from AAT Bioquest and used as received. Cyanine3 NHS ester was purchased from Lumiprobe and used as received.

### 2.2 Polymer characterization

After polymer synthesis ^1^H nuclear magnetic resonance (NMR) was performed using an Inova 300 to obtain number‐average molecular weight (M_n_). All samples were characterized in d‐chloroform. Absolute molecular weight and polydispersity were determined in the ASTRA software package (Wyatt Technology Corporation) after passing through two size exclusion chromatography columns (Resolve Mixed Bed Low DVB, ID 7.8 mm, Mw range 200–600,000 g/mol, Jordi Labs) in a mobile phase of N,N‐ Dimethylformamide (DMF) with 0.1 M LiBr at 35°C and a flow rate of 1.0 ml/min (Dionex Ultimate 3,000 pump, degasser, and autosampler (Thermo Fisher Scientific). Detection consisted of an Optilab T‐rEX (Wyatt Technology Corporation) refractive index detector operating at 658 nm and a HELEOS II light scattering detector (Wyatt Technology Corporation) operating at 659 nm. Dn/dc values for PEG and PLA were 0.0442 and 0.019 in the mobile phase were calculated using (dn/dc)_ab_ = (dn/dc)_a_(wt %)a + (dn/dc)_b_(wt %)_b_ after determining the dn/dc values for PEG and PEG–PLA polymers of known weight fractions (via ^1^H‐NMR spectroscopy) in the ASTRA software package by batch injection of three samples of known concentrations into an Optilab T‐rEX refractive index detector.

### 2.3 Rheological characterization

Rheological characterization was performed using a TA Instruments Discovery HR‐2 torque‐controlled rheometer fitted with a Peltier stage. All measurements were performed using a serrated 20‐mm plate geometry at 25°C. Dynamic oscillatory frequency sweep measurements were performed with a constant strain of 1% from 0.1 rad/s to 100 rad/s. Steady shear experiments were performed from 100 to 0.1 s^−1^. Yield stress values were found using a Herschel‐Bulkley fit to the flow sweep.

### 2.4 Preparation of HPMC‐C_12_

HPMC‐C_12_ was prepared according to previously reported procedures,^13^, ^14^ and the protocols will be briefly described here. HPMC (1.0 g) was dissolved in NMP (40 ml) by stirring at 80°C for 1 h before removing the heat. Once the solution cooled to room temperature, 1‐dodecylisocynate (105 mg, 0.5 mmol) and Hunig's base (catalyst, ~3 drops) were dissolved in NMP (5.0 ml). This solution was added dropwise to the reaction mixture, which was then stirred at room temperature for 16 h. This solution was then precipitated from acetone, decanted, re‐dissolved in water (~2 wt%) and placed in a dialysis tube for dialysis against water for 3–4 days. The polymer was lyophilized and reconstituted to a 60 mg/ml solution with sterile PBS.

### 2.5 Preparation of PEG–PLA

PEG–PLA was prepared as previously reported,^25^ and the protocols will be briefly described here. Monomethoxy‐PEG (5 kDa; 0.25 g, 4.1 mmol) and DBU (15 μl, 0.1 mmol; 1.4 mol% relative to LA) were dissolved in anhydrous DCM (1.0 ml). LA (1.0 g, 6.9 mmol) was dissolved in anhydrous DCM (3.0 ml) with mild heating. The LA solution was added rapidly to the PEG/DBU solution and was allowed to stir for 10 min. The reaction mixture was quenched and precipitated from a 1:1 solution of hexane and diethylether. The synthesized PEG–PLA was collected and dried under vacuum. Gel permeation chromatography (GPC) was used to verify that the molecular weight and dispersity of polymers meet our quality control (QC) parameters.

### 2.6 Preparation of TLR7/8a‐PEG–PLA

TLR7/8a‐PEG–PLA was prepared according to a literature report^26^ and the protocols will be briefly described here. Azide‐PEG–PLA was prepared by organocatalytic ring opening polymerization using N_3_‐PEG‐OH (0.5 g, 5 kDa, 100 μmol) in anhydrous DCM (2.0 ml) with DBU (30 μl, 30 mg, 0.20 mmol) which was added quickly to a stirring solution of LA (2.0 g, 13.9 mmol) in anhydrous DCM (6.0 ml). The solution was stirred for 10 min, after which 2 drops of acetic acid were added to quench the reaction, and the polymer was precipitated into a 1:1 mixture of hexanes and diethylether. The polymer was re‐dissolved in a minimal amount of acetone, and precipitated again in diethyl ether, and dried in vacuo. GPC was used to verify that the molecular weight and dispersity of polymers meet our QC parameters.

A 20 ml scintillation vial was charged with an alkyne‐functional derivative of our TLR7/8 agonist of interest, N‐(4‐((4‐amino‐2‐(ethoxymethyl)‐1H‐imidazo[4,5‐c]quinolin‐1‐yl)methyl)benzyl)‐3‐(prop‐2‐yn‐1‐yloxy)propenamide^26^ (14 mg, 30 μmol), the azide‐PEG–PLA (0.5 g, 20 μmol) species generate above, and NMP (4.0 ml). The reaction mixture was then sparged with nitrogen for 10 min. Next, a degassed solution (0.1 ml) of CuBr (3.7 mg/ml) and THPTA (16 mg/ml) was added. The reaction mixture was further sparged with nitrogen gas for 10 min. The reaction mixture was incubated for 16 h at room temperature and precipitated into diethylether in a 50 ml centrifuge tube to recover the polymer. The polymer was then dissolved in ethyl acetate and precipitated into diethyl ether, followed by collection and drying in vacuo. GPC was used to verify that the molecular weight was not altered by conjugation.

### 2.7 General preparation of PEG–PLA NPs, TLR7/8a NPs, and AF647 NPs

NPs were prepared as previously reported.^14^, ^16^ A 1 ml solution of PEG–PLA in DMSO (50 mg/ml) was added dropwise to 10 ml of water at room temperature under a high stir rate (600 rpm). NPs were purified by ultracentrifugation over a filter (molecular weight cut‐off of 10 kDa; Millipore Amicon Ultra‐15) followed by resuspension in water to a final concentration of 200 mg/ml. NPs were characterized by dynamic light scattering (DLS) to find the NP diameter and zeta potential (PEG–PLA NPs, 32 ± 4 nm, −28 ± 7 mV; TLR7/8a‐PEG–PLA NPs, 29 ± 3 nm, −10 ± 7 mV). AF647 NPs were prepared using a combination of PEG–PLA (25 mg) and unconjugated azide‐PEG–PLA (25 mg), then functionalized following purification by mixing azide‐functional NPs (500 μl, 20 wt%) with AF647‐DBCO (5 μl, 1 mg/ml).

### 2.8 General PNP hydrogel preparation

The 2:10 PNP hydrogel formulation contained 2 wt% HPMC‐C_12_ and 10 wt% PEG–PLA NPs in PBS. These gels were made by mixing a 2:3:1 weight ratio of 6 wt% HPMC‐C_12_ polymer solution, 20 wt% NP solution, and PBS. For TLR7/8a‐NP gels, the PEG–PLA NPs were made up of a mixture of TLR7/8a conjugated NP and non‐conjugated NP based on the desired dose of adjuvant. The solutions were mixed with an elbow mixer and loaded into a syringe.

### 2.9 FRAP analysis

PNP hydrogel formulations were made as stated above, each with a unique fluorescent component (FITC‐Dex, NP‐tethered AF647, rhodamine‐conjugated HPMC‐C_12_, His‐tagged hemagglutinin conjugated with HIS‐Lite‐Cy3 Bis NTA‐Ni Complex). FITC‐Dex of multiple sizes was used as fluorescent cargo: (a) 4 kDa, R_H_ = 14 Å, (b) 40 kDa, R_H_ = 45 Å, (c) 70 kDa, R_H_ = 60 Å, and (d) 250 kDa, R_H_ = 120 Å (R_H_ from Sigma product information). Gels were placed onto glass slides and imaged using a confocal LSM780 microscope. Samples were imaged using low intensity lasers to collect an initial level of fluorescence. Then a high intensity laser was focused on a region of interest (ROI) with a 25 μm diameter for 10 s in order to bleach a circular area. Fluorescence data was then recorded for 4 min to create an exponential fluorescence recovery curve. Samples were taken from different regions of each gel (*n* = 2–5). The diffusion coefficient was calculated according to the following equation^27^:

$$D=\gamma_{D}\left(\frac{\omega^{2}}{4\tau_{\frac{1}{2}}}\right),$$

where the constant γ_D_ = τ_1/2_/τ_D_, with τ_1/2_ being the half‐time of the recovery, τ_D_ the characteristic diffusion time, both yielded by the ZEN software, and *ω* the radius of the bleached ROI (12.5 μm).

The diffusivity of the HA antigen in PBS was calculated using the Stokes‐Einstein Law Equation for diffusion^28^ where k_B_ is Boltzmann's constant, T is temperature in Kelvin, η is solvent viscosity, and R is solute hydrodynamic radius:

$$D=\frac{k_{B}T}{6\mathrm{\pi \eta R}},$$

where the R of the HA antigen was taken to be 60 Å^29^ and the η for PBS was taken to be 0.8872 mPa.s at 25°C.^30^

### 2.10 IVIS measurement and analysis

PNP hydrogels were made as stated above using the 2:10, 2:5, 1:10, and 1:5 formulations. Each 100 μl gel contained 5 μg of fluorescent AF647‐conjugated NPs and 5 μg of Cyanine3‐conjugated HA. As the excitation and emission of these two dyes do not interfere with each other, experiments were conducted with both fluorescently labelled species. Cohorts of mice were injected with 100 μl of each formulation (*n* = 3) and imaged using the in vivo Imaging System (IVIS Lago) at multiple timepoints. For imaging, mice were anesthetized with isoflurane gas and imaged with an exposure time of 5 s for HA measurement (Cyanine3‐HA: excitation 540 nm, emission 570 nm) and 0.5 s for NP measurement (AF647‐NP: excitation 600 nm, emission 670 nm). The total flux of photons/s in region of interest (gel injection site) was quantified. To find t_50%_, the time at which each mouse's HA fluorescence intensity reached 50% of the initial value was calculated and the values of each cohort were averaged.

### 2.11 Vaccine formulations

The influenza vaccine contained a 5 μg dose of Influenza A H1N1 (A/California/04/2009) hemagglutinin (HA) (Sino Biological) and varied amounts of TLR7/8a, HPMC‐C12, and NPs. Consistent between all gel groups unless specifically altered: 2 wt% HPMC‐C_12_: 10 wt% NP, conjugated NPs have 10% TLR7/8a conjugated PEG–PLA and 90% unconjugated PEG–PLA, 20 μg TLR7/8a total dose. For the PNP hydrogels, the vaccine cargo was added at the appropriate concentration into the PBS component of the hydrogel before adding the polymer and NP solutions, as described above. For Alum (Invivogen) vaccines, the formulation was prepared according to the manufacturer's instructions with a 5 μg dose of HA.

### 2.12 Animal protocols

NIH guidelines for the care and use of laboratory animals (NIH Publication #85–23 Rev. 1985) have been observed. All animal studies were performed with the approval of Stanford Administrative Panel on Laboratory Animal Care.

### 2.13 Vaccination

C57BL/6 mice were purchased from Charles River and housed at Stanford University. Female mice between 6 and 10 weeks of age at the start of the experiment were used. The mice were shaved several days before vaccine administration and received a subcutaneous injection (100 μl administration volume) of gel or bolus vaccine on their backs under brief isoflurane anesthesia. Blood was collected from the tail vein at predetermined timepoints for survival studies.

### 2.14 Antibody concentration

Serum IgG antibody titers for the influenza vaccine were measure using an ELISA. Ni‐coated plates (Thermofisher) were coated with HA (Sino Biological) at 2.5 μg/ml in PBS for 1 h at 25°C and blocked with PBS containing 1% BSA for 1 h at 25°C. First, serum was diluted 1:250 and then four‐fold serial dilutions were performed up to 1:4,096,000 dilution. Titrations were added to plates and after 2 h at 25°C, goat–anti‐mouse IgG Fc‐HRP (1:10,000, Invitrogen, A16084) was added for 1 h at 25°C. Plates were developed with TMB substrate (TMB ELISA Substrate [High Sensitivity], Abcam). The reaction was stopped with 1 M HCl. The plates were analyzed using a Synergy H1 Microplate Reader (BioTek Instruments) at 450 nm. Endpoint titers were defined as the reciprocal of the highest serum dilution that gave an optical density above 0.1 and normalized to a pooled control used with each reading.

### 2.15 Statistical analysis

Comparisons between two groups were conducted by a two‐tailed Student's *t* test. One‐way ANOVA test with a Tukey's multiple comparisons test was used for comparison across multiple groups. Statistical analysis was run using GraphPad Prism 7.04 (GraphPad Software). Statistical significance was considered as p < 0.05.

## 3 RESULTS

Our previous work has shown that PNP hydrogels can be loaded with vaccine components for injection and provide extended co‐delivery of physicochemically disctinct subunit vaccine cargo comprising ovalbumin and poly(I:C). These hydrogels are formed rapidly by mixing aqueous solutions of dodecyl‐modified hydroxypropylmethylcellulose (HPMC‐C_12_) with biodegradable polymeric NPs composed of poly(ethylene glycol)‐b‐poly(lactic acid) (Figure 1(a)).^14^, ^16^, ^31^, ^32^, ^33^, ^34^, ^35^ The HPMC‐C_12_ polymer adsorbs onto the PEG–PLA NPs, forming multivalent and dynamic non‐covalent interactions that constitute the physical cross‐links of the hydrogel structure (Figure 1(a)).^36^ The simplicity of the PNP hydrogel synthesis via simple mixing, performed here with an elbow mixer or spatula, allows for ease of production and potential for large scale fabrication of PNP hydrogels with consistent mechanical and structural properties.^17^, ^37^ This platform is also highly tunable in physical properties by changing the ratio of HPMC‐C_12_ to NP to an aqueous solution.

In this work we sought to augment the PNP hydrogel delivery platform for sustained co‐delivery of the influenza antigen hemagglutinin (HA), the most common antigen in standard influenza subunit vaccines, with a potent TLR7/8a adjuvant that has previously been shown to elicit strong titers against HA and has demonstrated promise for clinical translation in influenza vaccination.^38^ TLR‐7/8a adjuvant systems have been shown to enhance CD8 T‐cell responses in cancer vaccination models,^39^ induce an influx of migratory DCs to the LN, and increase DC antigen uptake during vaccine responses,^40^ as well as increase antibody responses to HIV vaccines in non‐human primates.^41^

To facilitate co‐delivery of the large HA antigen (~80 kDa) and the small molecule TLR7/8a adjuvant (314 Da), we tethered TLR7/8a to the NPs which constitute a structural motif within the PNP hydrogels of our system, according to literature reports (Figure 1(b)).^24^, ^26^ The TLR7/8a molecule was conjugated to PEG–PLA using copper‐catalyzed “click” chemistry of an alkyne‐modified TLR7/8a with azide‐terminated PEG–PLA (Supplementary Figure S1). The TLR7/8a‐conjugated PEG–PLA was then physically mixed with standard PEG–PLA at predetermined ratios and nanoprecipitated into water to create TLR7/8a‐NPs with a controlled density of TLR7/8a molecules on the corona of the NPs. Two different populations of TLR7/8a‐NPs were fabricated using either a mixture of 10 wt% TLR7/8a‐conjugated PEG–PLA with 90 wt% standard PEG–PLA, or a mixture of 50 wt% of both TLR7/8a‐conjugated PEG–PLA and standard PEG–PLA. The density of TLR7/8a molecules on the NPs is denoted as 10% or 50% based on the loading of TLR7/8a‐PEG–PLA used in the TLR7/8a‐NP synthesis.

We hypothesized that differences in the physical properties of the hydrogels would impact vaccine cargo diffusivity and lead to differences in the observed humoral immune responses to hydrogel‐based immunization (Figure 1(c)). We modulated gel composition by changing the ratio of HPMC‐C_12_ to NP to an aqueous buffer solution during mixing (Figure 1(c)(i)). A solution of 2 wt% HPMC‐C_12_: 10 wt% NP: 88 wt% saline solution is represented throughout as a “2:10” gel formulation, while one consisting of 1 wt% HPMC‐C_12_: 5 wt% NP: 94 wt% saline solution is denoted “1:5”. The distribution of TLR7/8a within the hydrogels could be modulated by changing the amount of TLR7/8a displayed on the TLR7/8a‐NPs (i.e., 10% or 50% display density) while keeping the overall TLR7/8a dose within the hydrogels constant by simply mixing TLR7/8a‐NPs with standard PEG–PLA NPs (Figure 1(c)(ii)). Further, the dose of TLR7/8a molecules within the hydrogels could be controlled to see how low of a TLR7/8a dose can yield efficacious vaccine responses with the PNP hydrogel platform (Figure 1(c)(iii)).

**FIGURE 2:**
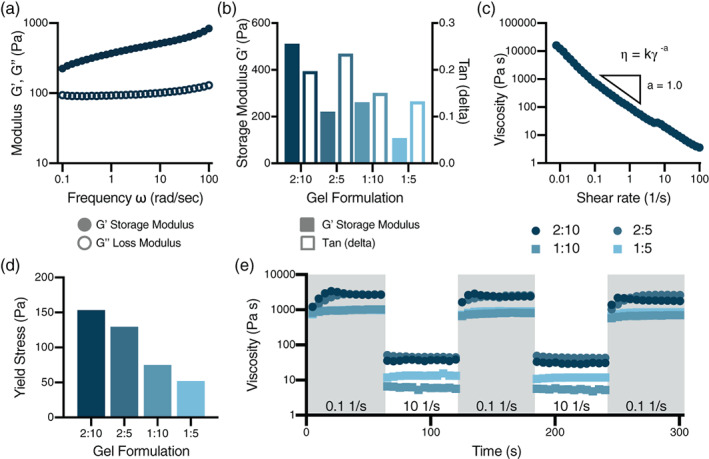
Rheological properties of varied composition hydrogels. (a) Sample data from 2:10 gel frequency‐dependent oscillatory shear experiments indicates that these materials exhibit solid‐like properties with the storage modulus (G') greater than the loss modulus (G") at all frequencies tested at 1% strain. (b) Plotting data taken at a single representative angular frequency (ω = 10 rad/s, strain = 1%) shows a decreasing trend in storage moduli as gels decrease in solids content. Tan(δ) values less than 1 indicate that all gels have G' > G” and are therefore solid‐like at low stresses. (c) Example shear‐dependent viscosity data of the 2:10 gels from a shear rate sweep showing decreasing viscosity with increasing shear rate and a large power‐law shear‐thinning index “a”. (d) Yield stresses determined from the shear rate sweep through fitting of the data with the Herschel‐Bulkley model show a decreasing trend as gels decrease in solids content. (e) Step‐shear measurements whereby the materials are subjected to cycles of low and high shear rates (0.1 s^−1^, 10 s^−1^) in 60 s steps. These assays demonstrate the gels' ability to shear‐thin and self‐heal under flow conditions mimicking the process of injection. The viscosity of all PNP hydrogels decreased around two orders of magnitude at the high shear rates and rapidly recovered the mechanical propertie through multiple cycles

The viscoelastic properties of four PNP hydrogel formulations comprising differing polymer and NP compositions were characterized using rheological techniques. Frequency‐dependent oscillatory shear experiments were performed in the linear viscoelastic regime on each sample to measure the storage (G') and loss (G") moduli. All gels demonstrated solid‐like properties, with G' greater than G" at all frequencies evaluated, shown in the representative case of the 2:10 gel (Figure 2(a), Supplementary Figure S2). Plotting the mechanical properties at a single representative angular frequency (ω = 10 rad/s) shows a decreasing trend in both storage and loss moduli as gels decrease in network density, consistent with previous observations for similar materials (Figure 2(b)).^31^ A flow sweep evaluating the shear‐rate dependence of the materials was performed to examine the gels' viscosity and yield stress behavior (Figure 2(c),(d)). All samples exhibited decreasing viscosity with increasing shear rate, shown in the representative case of the 2:10 gel (Figure 2(c), Supplementary Figure S3). All gels exhibited a shear‐thinning index, a, from the relationship $\eta =k{\dot{\gamma }}^{-a}$ of around 1, indicating a high degree of shear‐thinning that is necessary for injectability.^42^, ^43^, ^44^ Yield stresses, which are critical for maintenance of a robust depot in the body following administration,^14^ were determined through a fit to the Herschel‐Bulkley model and showed a decreasing trend as gels decreased in network density (Figure 2(d)). To compare the gels' abilities to shear‐thin and rapidly self‐heal, step‐shear measurements were conducted by applying a stepwise change between low and high shear rates (0.1 s^−1^ and 10 s^−1^, respectively). Following removal of high shear rates, all of the formulations rapidly recovered their mechanical properties. This behavior was observed through multiple cycles of shearing and self‐healing, suggesting that the decrease in viscosity upon shearing is driven by rupture of the non‐covalent PNP crosslinking interactions forming the hydrogel network rather than any covalent bonds (Figure 2(e)). These mechanical properties indicate that all PNP hydrogel formulations can be injected through a syringe needle, then rapidly form a solid‐like depot locally at the injection site.

**FIGURE 3:**
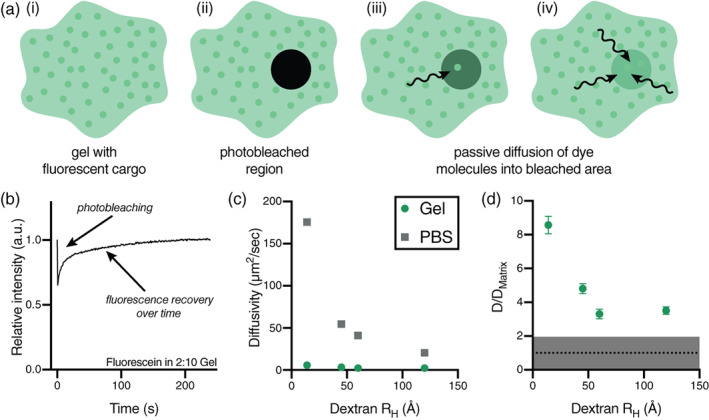
Fluorescence recovery after photobleaching experiments characterize 2:10 hydrogel mesh size. FRAP experiments were performed on the 2:10 formulation hydrogel using multiple sizes of FITC‐Dex as fluorescent cargo: (i) 4 kDa, R_H_ = 14 Å, (ii) 40 kDa, R_H_ = 45 Å, (iii) 70 kDa, R_H_ = 60 Å, and (iv) 250 kDa, R_H_ = 120 Å. (a) A defined circular region of each gel is bleached via exposure to intense light and passive diffusion of dye‐conjugated molecules into the region causes local fluorescence recovery. (b) Fluorescence recovery over time generates a curve from which the diffusivity of the fluorescent components can be calculated. (c) The gel network causes significantly slowed cargo diffusion compared with the diffusivities of FITC‐Dex in PBS (calculated with the Stokes‐Einstein equation). (d) Comparison of the FITC‐Dex diffusivity values with the diffusivity of the polymer matrix (taken as the diffusivity of the HPMC‐C_12_ component, D_matrix_ = 0.68 ± 0.11 μm^2^/sec) shows that cargo diffusion decreases with increasing cargo size, reaching a plateau at a hydrodynamic radius of around 60 Å, after which entrapped cargoes diffuse at similar rates

To investigate the effect of cargo size on molecular diffusion through the PNP hydrogel, fluorescence recovery after photobleaching (FRAP) experiments were performed on the 2 wt% HPMC‐C_12_ + 10 wt% NP (2:10) formulation gel using fluorescein isothiocyanate‐functional dextran (FITC‐Dex) molecules of various molecular weights and hydrodynamic radii as fluorescent cargo (Figure 3(a)). Multiple hydrogels were prepared with each unique FITC‐Dex. In these assays, a defined circular region of each gel was bleached via exposure to intense light and the recovery of the fluorescence in the bleached region was measured over time to monitor the passive influx of FITC‐Dex species from outside the bleached region. The curve of fluorescence recovery over time (Figure 3(b)) was used to calculate the diffusivity of each component. The PNP gel network significantly slowed cargo diffusion when compared with the diffusivities of FITC‐Dex in phosphate buffered solution (PBS), which is a standard mode of delivery for subunit vaccines (Figure 3(c)), highlighting the effectiveness of the PNP hydrogel platform for sustained cargo delivery.

When compared to the diffusivity of the polymer matrix (determined to be the diffusivity of the HPMC‐C_12_ component of the hydrogel), we observed that cargo diffusivity decreased with cargo size until a plateau was reached at a hydrodynamic radius of around 60 Å, after which cargoes diffuse at a similar rate (Figure 3(d)), suggesting that the mesh size of the 2:10 hydrogel network is approximately 60 Å. Cargoes with a radius smaller than the mesh size will follow a curve of decreasing diffusivity with increasing size,^45^, ^46^ while cargoes with larger radii will be immobilized in the polymer matrix and diffuse at a rate close to the self‐diffusivity of the polymer network of the hydrogel itself.^14^, ^47^

**FIGURE 4:**
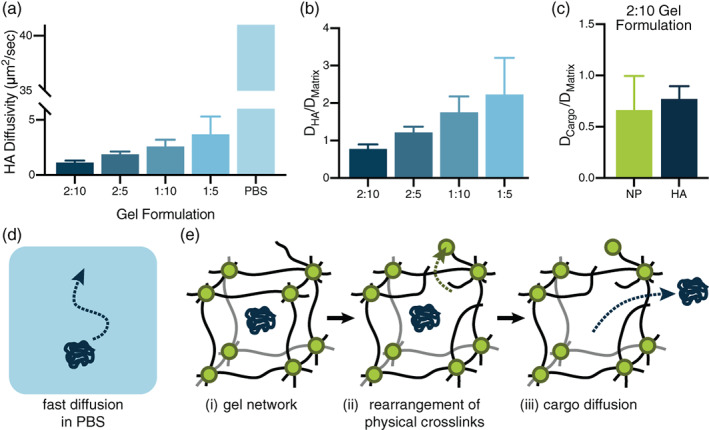
Hemagglutinin diffusion in varied hydrogel formations. (a), All gel formulations significantly slowed HA diffusion compared to the antigen's diffusivity in PBS (PBS diffusivity calculated with the Stokes‐Einstein equation). The gel formulation with the largest weight percent of polymer and NPs exhibited the lowest HA diffusivity, with diffusivity increasing as polymer and NP components were lessened. (b), Normalization of HA diffusivities in each formulation by HPMC‐C12 matrix diffusivity shows that HA diffusion is primarily dictated by the diffusion of the matrix. (c), Normalization of the diffusivity values of both the HA and NP components by the 2:10 hydrogel matrix diffusivity (D_HA_/D_matrix_), yields values close to 1, indicating that these components are both immobilized in the hydrogel mesh and diffuse at the same rate as the matrix itself. (d), Diagram illustrating fast diffusion of vaccine components in PBS. (e), Diagram of the PNP hydrogel network (i) entrapping HA cargo, revealing how (ii) the hydrogel network must dynamically rearrange for (iii) diffusion of entrapped vaccine components to occur

The diffusivity of the most clinically relevant influenza antigen hemagglutinin (HA) was studied through FRAP experiments in the four distinct PNP hydrogel formulations. All gel formulations significantly slowed HA diffusion compared to the antigen's diffusivity in PBS (Figure 4(a), Supplementary Figure S4). Between gel samples, the formulation with the highest mass loading of hydrogel components exhibited the lowest HA diffusivity, with diffusivity increasing as polymer and NP components decreased. These data show that the 1:5 gel has a larger mesh size than the other formulations, allowing for a commensurate increase in the HA diffusivity and thus vaccine component delivery. When normalized by the diffusivity of the polymer matrix measured in each formulation, the diffusivity of the HA is shown to be primarily dictated by the matrix diffusivity, as all values of D_HA_/D_Matrix_ are closer to 1 than the non‐normalized D_HA_ values (Figure 4(b)). The 2:10 gel was used as a model for comparing HA and NP diffusivity in the gel to study the potential of the PNP hydrogel network to realize sustained co‐delivery of the antigen and adjuvant species (Figure 4(c)). It was found that NPs and HA have a similar diffusivity in the gel and normalization with the diffusivity of the polymer matrix (D/D_matrix_) yields a value close to 1 for both components (Figure 4(c)). These data suggest that diffusion of both vaccine components is again dictated by the self‐diffusion of the hydrogel matrix. These FRAP experiments show that while the HA antigen exhibits high diffusivity in PBS (Figure 4(d)), both the HA and NP‐tethered vaccine components require rearrangement and self‐diffusion of the PNP hydrogel network to diffuse (Figure 4(e), Table S1), enabling sustained co‐delivery of these components despite their physicochemical differences.

**FIGURE 5:**
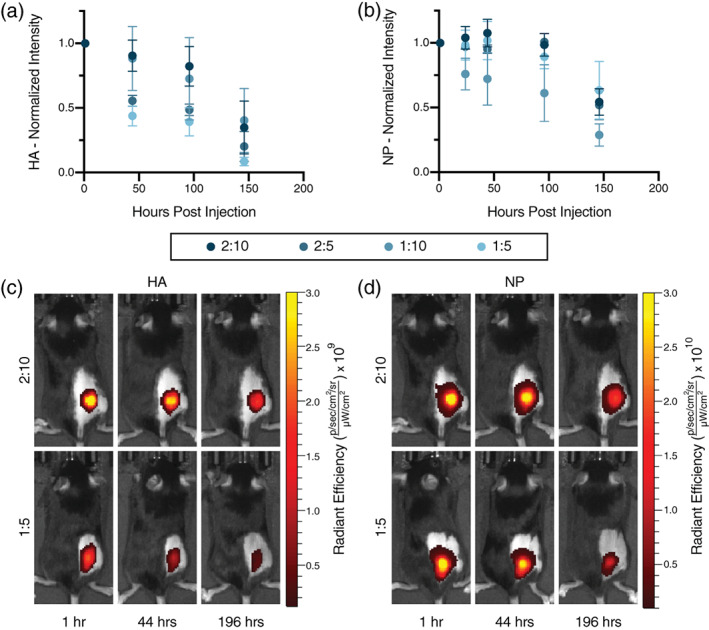
In vivo HA and NP tracking. IVIS measurements were performed on mice injected with previously tested vaccine hydrogels of the 2:10, 2:5, 1:10, and 1:5 formulations containing fluorescently tagged HA and NPs (*n* = 3). (a) Total intensity of Cy3‐tagged HA was measured post‐injection, with signal from HA in the weakest 1:5 gel dropping the most quickly. (b) Total intensity of AF647‐tagged NPs, representing adjuvant carrying NPs, demonstrating that stiffer hydrogel formulations retain their NPs and associated depot structure over longer time‐frames. Representative timepoints in the strongest (2:10) and weakest (1:5) hydrogel formulations demonstrate the ability of a stronger gel to retain (c) HA and (d) adjuvant carrying NPs for a longer duration than the weakest formulation

To measure the kinetics of release of each of the vaccine components in vivo following administration in PNP hydrogels, various hydrogel formulations (2:10, 2:5, 1:10, and 1:5) containing Cy3‐tagged HA and AF647‐tagged NPs were administered subcutaneously via transcutaneous injection to C57BL/6 mice (*n* = 3 per group). Intravital epifluorescence imaging was performed at multiple timepoints post‐injection using an In vivo Imaging System (IVIS) to quantify the amount of HA or NP remaining at the injection site over time. Interestingly, these measurements revealed that each hydrogel formulation exhibited similar release timeframes for both the HA antigen and the adjuvant NPs. The weakest gel formulation, PNP 1:5, released its HA antigen cargo more quickly than all of the stiffer hydrogel formulations (Figure 5(a)). The initial 50% of the entrapped HA was released from each hydrogel formulation in the following timeframes: (a) 1:5 reached t_50%_ in 57 ± 30 hrs, (b) 1:10 reached t_50%_ in 124 ± 50 hrs, (c) 2:5 reached t_50%_ in 86 ± 20 hrs, and (d) 2:10 reached t_50%_ in 132 ± 20 hrs. Moreover, the 1:5 hydrogel formulation also released its NPs much faster than all of the other hydrogel formulations (Figure 5(b)). Representative timepoints for the stiffest (2:10) and weakest (1:5) hydrogel formulations demonstrate the ability of stiffer depot material to retain HA (Figure 5(c)) and adjuvant‐conjugated NPs (Figure 5(d)) for a longer duration. These data suggest that stiffer hydrogels maintain their structure and retain their vaccine cargo over longer timeframes, likely enabling prolonged immune cell recruitment to the injection site.

**FIGURE 6:**
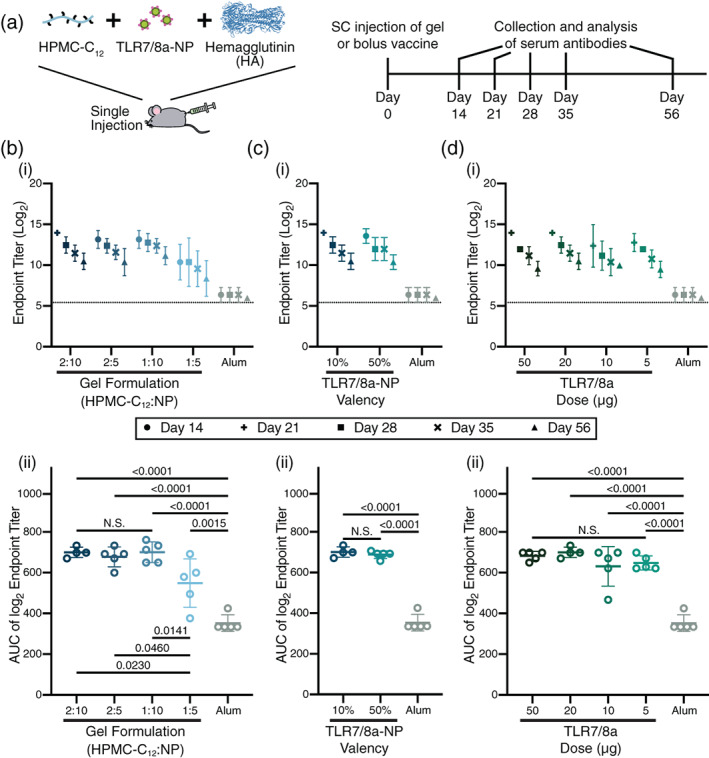
Hemagglutinin diffusion in varied hydrogel formations. (a) Mice were vaccinated subcutaneously with a single administration of HA (5 μg) in different formulations of PNP hydrogels comprising TLR7/8a‐NPs or an alum bolus control. Serum antibodies were collected and analyzed via ELISA at the indicated timepoints. All experimental gel groups performed significantly better than the bolus Alum control (b‐d; *N* = 5). (b) (i) Total IgG endpoint titers over time following immunization with HA entrapped in various hydrogel formulations comprising TLR7/8a‐NPs (10% valency; 20 μg total TLR7/8a dose) indicating that these slow release gels yield more potent and durable antibody responses than Alum. (ii) AUC calculation of endpoint titers over time indicated that 2:10, 1:10, and 2:5 hydrogel formulations all performed similarly, while the 1:5 hydrogel formulation exhibited lower total potency. (c) (i) Total IgG endpoint titers over time and (ii) AUC of endpoint titer over time following immunization with HA entrapped in gel formulations comprising various TLR7/8a‐NP valency (10% or 50% with a 20 μg total TLR7/8a dose). (d) (i) Total IgG endpoint titers over time and (ii) AUC of endpoint titer over time following immunization with HA entrapped in PNP hydrogels comprising various doses of TLR7/8a‐NP (10% valency; total TLR7/8a doses equal to 50, 20, 10, or 5 μg) demonstrating no significant difference in antibody response with TLR7/8a dose

We then evaluated the impact of slow PNP hydrogel‐based influenza vaccination on humoral immune responses. Mice (C57BL/6) were immunized subcutaneously with a single administration of HA encapsulated in different formulations of PNP hydrogels, PNP hydrogels comprising various concentrations of TLR7/8a adjuvant conjugated to TLR7/8a‐NPs, and PNP hydrogels comprising TLR7/8a‐NPs of different TLR7/8a valency at a single TLR7/8a dose (Figure 6(a)). These experimental groups were compared against HA administered with Alum, a clinically relevant adjuvant, in a PBS bolus. Each mouse received the same amount of HA (5 μg). A single administration was evaluated in these experiments because the standard clinical influenza vaccine is given once annually. Generally, all hydrogel‐based experimental groups performed significantly better than the Alum control, regardless of the formulation, indicating that slow co‐delivery of subunit vaccine components improves humoral immune responses (Figure 6(b)‐(d)).

We first examined the impact of PNP hydrogel formulation on antibody responses (Figure 6(b)). Each of the four different PNP hydrogel formulations described above were loaded with HA and TLR7/8a‐NPs with a valency of 10% and a 20 μg TLR7/8a total dose. We reported previously that the strongest gel formulation, 2:10, persists in the subcutaneous space for roughly 4 weeks while the weakest gel, 1:5, persists for only about 2 weeks.^14^ Coupled with the diffusivity data described above, the stiffer 2:10 gels were expected to provide the longest duration of vaccine release. Evaluation of total IgG endpoint titers over time indicated that all hydrogels performed better than the Alum control (Figure 6(b)(i)). The 2:10, 1:10, and 2:5 hydrogel formulations all performed similarly, and performed better than the 1:5 hydrogel formulation. Assessment of the area under the curve (AUC) of the IgG titers indicated that longer‐term vaccine exposure from the stiffer PNP hydrogel formulations led to higher overall vaccine potency and durability (Figure 6(b)(ii)). These observations are consistent with previous reports on slow vaccine delivery with PNP hydrogels,^14^ but suggest that a threshold for exposure time‐frame exists, beyond which slower vaccine delivery has a negligible added benefit. Based on these results, we chose to conduct all further studies using the 2:10 PNP hydrogel formulation.

We then sought to evaluate the effect of distribution of TLR7/8a throughout the gel at a single TLR7/8a dose (20 μg) using TLR7/8a‐NPs with a 10% TLR7/8a valency and 50% TLR7/8a valency, mixed with standard PEG–PLA NPs to achieve the same TLR7/8a dose. This study was conducted to understand whether have the adjuvant molecules more densely gathered adjuvant on the 50% TLR7/8a‐NPs or more spread out throughout the hydrogel on more particles in the case of the 10% TLR7/8a‐NPs potentiated better overall immune activation. Evaluation of the total IgG endpoint titers over time indicated no difference between these two methods of TLR7/8a delivery (Figure 6(c)[i,ii]), suggesting that the primary design consideration is the overall TLR7/8a dose. For this reason, we selected the 10% TLR7/8a‐NPs for the remaining vaccine studies.

To evaluate the ability of slow PNP hydrogel‐based vaccine delivery to enhance adjuvant potency, we varied the total dose of TLR7/8a from 50–5 μg. Similar to the previous set of experiments, decreasing the total dose of TLR7/8a had no significant effect on the observed antibody responses (Figure 6(d)[i,ii]). These data indicate that the PNP hydrogel platform drives potent vaccine responses even with low doses of TLRa, enabling significant dose sparing of the TLR7/8a adjuvant without surrendering overall vaccine efficacy at producing a potent and durable antibody response.

## 4 DISCUSSION

In this work we have designed an easily administered slow‐delivery influenza vaccine platform that elicits a robust and durable antibody response. Our injectable PNP hydrogel allows spatiotemporal control over antigen and adjuvant presentation to the immune system by prolonging co‐delivery of influenza hemagglutinin antigen and a potent TLR7/8a adjuvant. This work highlights that the PNP hydrogel platform has the potential to support many different combinations of antigen and adjuvant. We show that the mechanical and structural characteristics of the hydrogel, as well as cargo diffusivity, can be modulated through simple alteration of the gel formulation. We show a relationship between hydrogel physical properties and the resulting immune response to immunization by inoculating mice with hydrogel‐based vaccines of varied compositions.

Rheological analysis of our materials exhibited their injectability, self‐healing properties, and changing network strength with formulation modulation. All gel formulations displayed solid‐like properties and robust yield stresses as well as a large dependence of the viscosity on the shear rate. This high degree of shear‐thinning, coupled with rapid recover of the mechanical properties following shear, enable facile injectability.^14^, ^15^ These mechanical properties demonstrate that the PNP hydrogels yield under the high pressure delivered by syringe injections and flow smoothly through the needle into the subcutaneous space, where the materials rapidly reforms into a solid‐like depot for extended cargo delivery. Gels made with less polymer and/or NP components exhibited lower moduli and yield stresses, showing that we are able to tune our system to make stronger or weaker hydrogels.

Cargo diffusivity within the gel was shown to be tunable through modulation of the hydrogel composition using FRAP measurements of various fluorescent dye‐labeled molecules. We demonstrate that the PNP hydrogel has a mesh size above which larger particles will become immobilized in the polymer network and diffuse at a constant rate. In a 2:10 gel, both HA and TLR7/8a‐NPs diffuse at a similar rate, allowing for sustained co‐delivery. Changing the gel composition allows HA to diffuse at incrementally faster rates, though all gels vastly slow HA diffusivity compared to HA diffusivity in a standard saline solution. These observations demonstrate that the PNP hydrogel matrix is able to provide sustained co‐delivery of two distinct chemical moieties.

When administered in mice, hydrogel‐based vaccines demonstrated enhancements in the magnitude and duration of antibody responses compared to alum, a widely used clinical adjuvant system. We found that stiffer hydrogel formulations resulted in the greatest improvements to the antibody response, suggesting that slower diffusion time potentiates a stronger extended humoral response. Yet, the stiffest hydrogel formulations all performed similarly, highlighting that a threshold of time frame for vaccine exposure may exist beyond which slower diffusion has negligible added benefit. In a previous study, our PNP gel platform comprising an NP‐tethered TLR7/8a adjuvant exhibited a 2 orders of magnitude improvement in titer response compared to a PNP gel containing only antigen and no adjuvant, indicating the necessity for inclusion of an adjuvant within the hydrogel platform.^14^ Further study in this current work reveals that variation in the dose of TLR7/8a delivered within these hydrogel‐based vaccines had no significant effect on the humoral immune response, highlighting that the PNP hydrogel platform enables significant dose sparing of TLR7/8a adjuvant without compromising vaccine efficacy. TLR7/8a dose sparing can be useful from a translational perspective for mitigating toxicity while maintaining efficacy, as well as reducing costs.

Further investigation into the diffusive properties of our vaccine components in vivo revealed that HA and NPs are retained for significantly longer amounts of time in the three stiffest hydrogels. This observation suggests that there is a threshold timeframe for vaccine exposure whereby the three stiffest formulations performed similarly in eliciting potent antibody responses. These three hydrogels likely persist for a sufficiently long time to allow for prolonged recruitment of immune cells to the injection site as well as a longer time for immune cells to interact with the entrapped antigen and adjuvant. Furthermore, the similar release timescales observed for both HA and NPs in vivo corroborated our in vitro diffusivity measurements, indicating that our hydrogel provides robust temporal and spatial control over vaccine cargo delivery.

We have previously demonstrated that slow delivery of a model vaccine comprising OVA and poly(I:C) leads to the generation of a local inflammatory niche and prolonged GCs reactions that improve overall antibody responses.^14^ Here we have shown that influenza vaccines comprising TLR7/8a‐NPs and HA can be delivered over prolonged time frames within PNP hydrogels, significantly improving humoral immune responses over clinical adjuvants. These observations suggest that creation of a local inflammatory niche to prolong interactions between TLR7/8a, HA, and innate immune cells may improve antigen processing and presentation, giving rise to the enhanced responses observed in these studies. Future research into the role of the adjuvant identity and time‐scale of vaccine exposure can provide useful design criteria for next‐generation influenza vaccines provided potent, durable, and broad immunity.

## 5 CONCLUSIONS

In conclusion, here we show that the self‐assembled, injectable PNP hydrogel platform can be adapted for the sustained delivery of subunit influenza vaccines to improve the potency and durability of humoral immune responses. By conjugating a small molecule TLR7/8 agonist adjuvant to the NP component of the hydrogels, we showed the ability of this hydrogel platform to enable sustained co‐delivery of antigens and adjuvants of interest in a truly modular fashion, creating better antibody responses while also enabling significant adjuvant dose sparing. With this tool we can better understand how kinetics of vaccine exposure can improve vaccine responses. This study suggests that our PNP hydrogel represents a highly tunable platform for effectively manipulating the humoral immune response for any subunit vaccine of interest, potentially aiding in the development of next‐generation vaccines against challenging pathogens such as influenza.

## CONFLICTS OF INTEREST

Gillie A. Roth, Emily C. Gale, Anton A. A. Smith, and Eric A. Appel are listed as inventors on patent applications (63/025,845; 62/739,587) describing the technology reported in this manuscript.

## AUTHOR CONTRIBUTIONS

Olivia M. Saouaf, Gillie A. Roth, and Anton A. A. Smith designed the experiments. Olivia M. Saouaf, Gillie A. Roth, Ben S. Ou, Anton A. A. Smith, and Vittoria C.T.M. Picece performed the experiments. Olivia M. Saouaf analyzed the data. Gillie A. Roth, Anthony C. Yu, Emily C. Gale, Abigail K. Grosskopf, and Eric A. Appel supervised the experimental techniques. Abigail K. Grosskopf and Eric A. Appel reviewed the manuscript. All authors were involved in writing the paper and have approved the final manuscript.

## Supporting information


**Appendix S1**: Supporting Information

## Data Availability

All data supporting the results in this study are available within the Article and its Supplementary Information. The broad range of raw datasets acquired and analyzed (or any subsets of it), which for reuse would require contextual metadata, are available from the corresponding author on reasonable request.
