# Supplementary material for: Modulation of injectable hydrogel properties for slow co‐delivery of influenza subunit vaccine components enhance the potency of humoral immunity
Source: J Biomed Mater Res A. 2021 May 6;109(11):2173–86. doi: 10.1002/jbm.a.37203 (PMC8518857; doi:10.1002/jbm.a.37203)
Supplement: Supplementary file 1 — Appendix S1: Supporting Information [file JBM-109-2173-s001.docx]

**SUPPLEMENTARY INFORMATION**

**Modulation of injectable hydrogel properties for slow co-delivery of influenza subunit vaccine components enhance the potency of humoral immunity**

Olivia M. Saouaf^1^, Gillie A. Roth^2^, Ben S. Ou^2^, Anton A. A. Smith^1^, Anthony C. Yu^1^, Emily C. Gale^3^, Abigail K. Grosskopf^4^, Vittoria C.T.M. Picece^1,5^, Eric A. Appel^1,2,6,7,8*^

^1^Department of Materials Science & Engineering, Stanford University, Stanford, CA 94305, USA

^2^Department of Bioengineering, Stanford University, Stanford, CA 94305, USA

^3^Department of Biochemistry, Stanford University School of Medicine, Stanford, CA 94305, USA

_­­­_^4^Department of Chemical Engineering, Stanford University, Stanford, CA 94305, USA

^5^Department of Chemistry & Applied Biosciences, ETH Zürich, Zürich 8093, Switzerland

_­­­_^6^Institute for Immunity, Transplantation & Infection, Stanford University School of Medicine, Stanford, CA 94305, USA

^7^ChEM-H Institute, Stanford University, Stanford, CA 94305, USA

^8^Department of Pediatrics - Endocrinology, Stanford University School of Medicine, Stanford, CA 94305, USA

* corresponding author: eappel@stanford.edu

Table of Contents

[Supplementary Figures 3](#_Toc53474798)

[Figure S1. Synthesis of TLR7/8 agonist PEG-PLA conjugate 3](#_Toc53474799)

[Figure S2. Rheological frequency sweeps of each gel formulation 4](#_Toc53474800)

[Figure S3. Viscosity measurements from shear rate sweeps of each gel formulation. 5](#_Toc53474801)

[Figure S4. Fluorescence recovery curves of fluorescently tagged hemagglutinin in gels 6](#_Toc53474802)

[Figure S5. IVIS images of fluorescently tagged vaccine cargo in mice 7](#_Toc53474802)

[Supplementary Tables 8](#_Toc53474803)

[Table S1. Cargo and Polymer Diffusivities in 2:10 PNP hydrogel 8](#_Toc53474804)

References 9

**Supplementary Figures**


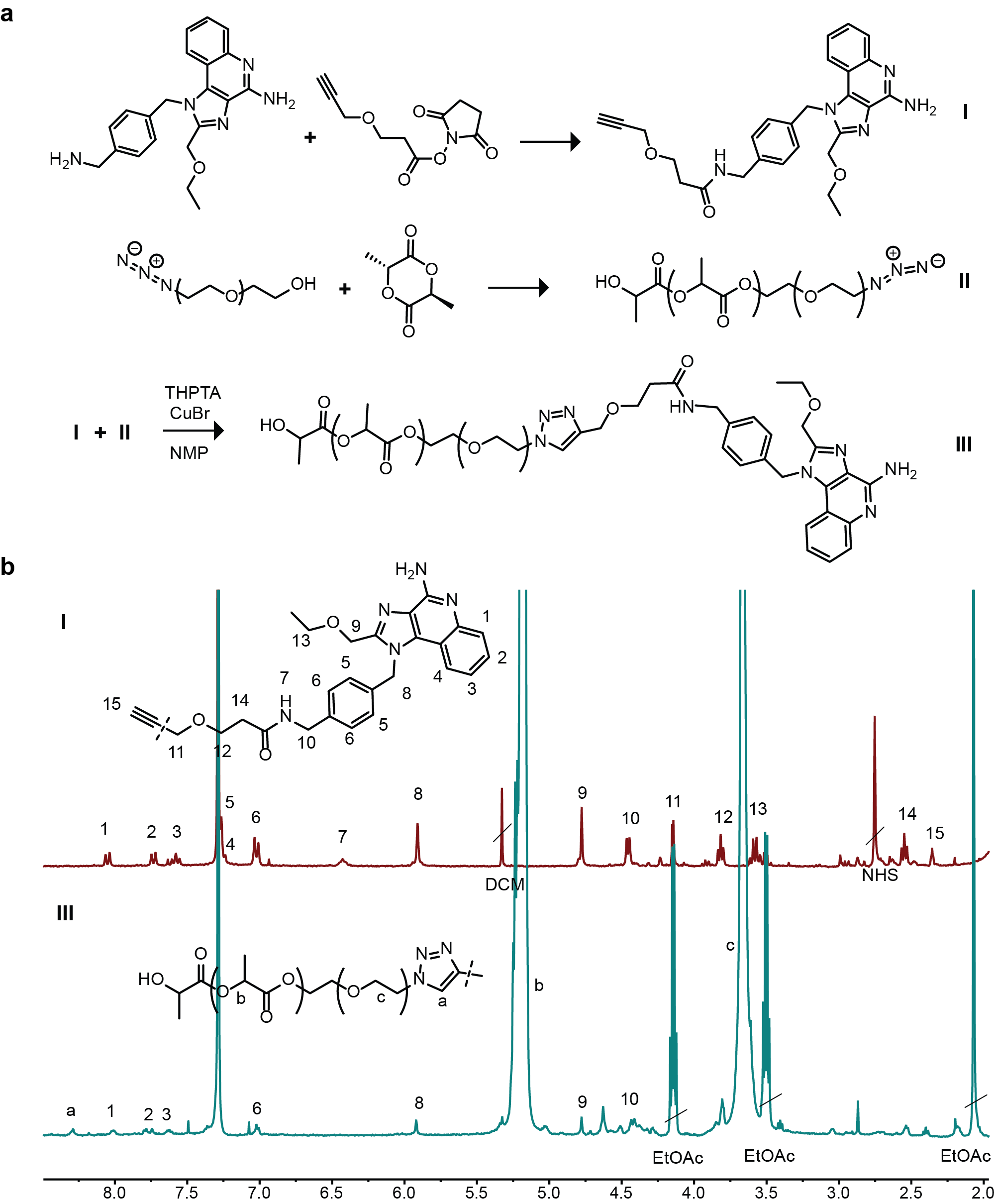


**Figure S1 | Synthesis of TLR7/8a-PEG-PLA conjugate. a**, NHS coupling of TLR7/8 agonist to alkyne (I), followed by coupling to azide-terminated PEG-PLA (II) to make TLR7/8a-PEG-PLA with the TLR7/8a presenting on the hydrophilic PEG terminus of the block copolymer (III). **b**, ^1^H-NMR spectrum of TLR7/8a alkyne (I) stacked with TLR7/8a-PEG-PLA conjugate (III). Broadening of peaks corresponds to the TLR7/8a molecule and the emergence of the triazole proton, a, along with disappearance of terminal alkyne proton, 15, confirms conjugation and the formation of III.

**
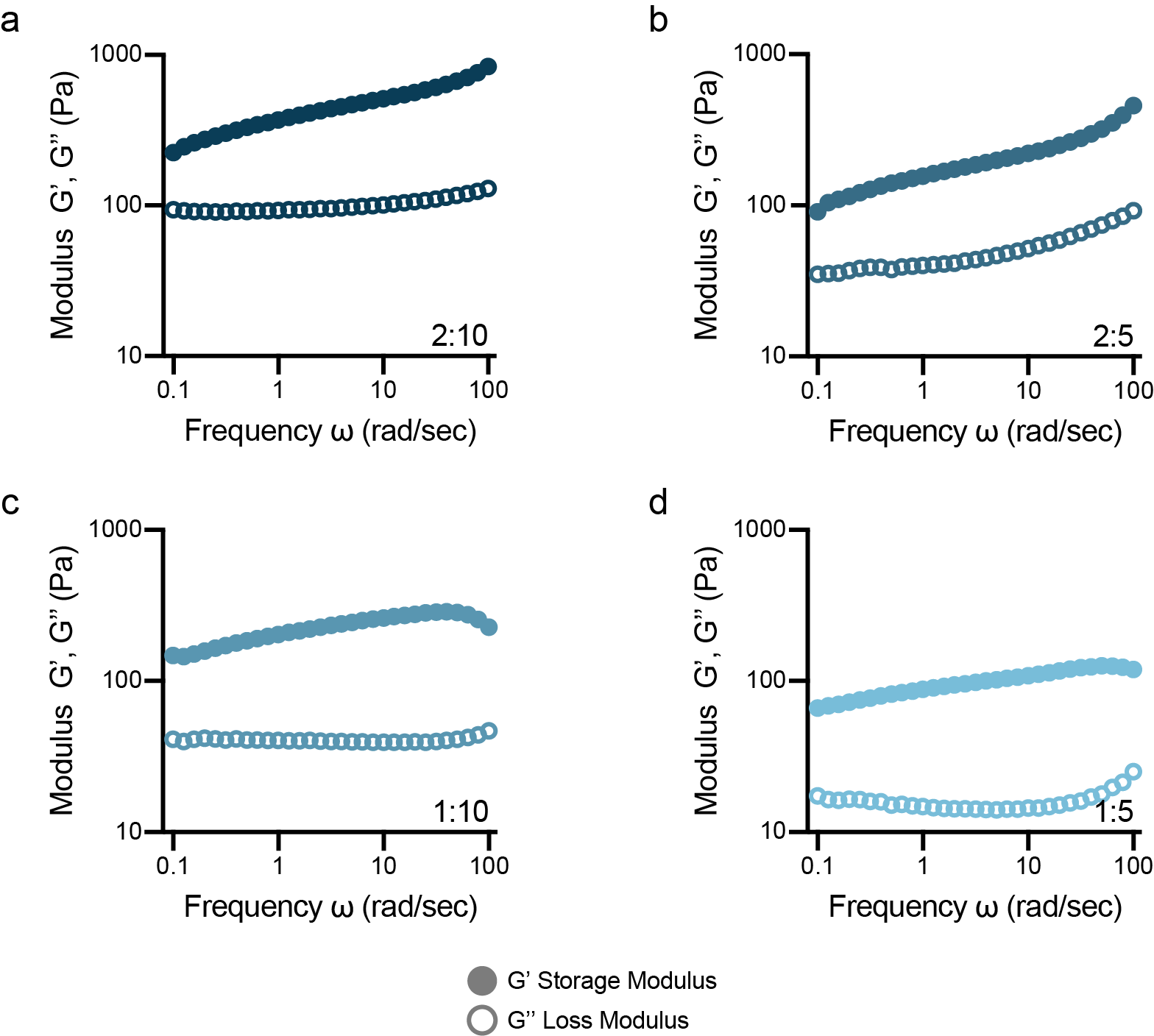
**

**Figure S2 | Rheological frequency sweeps of each gel formulation.** Frequency sweeps from low (ω = 0.1 rad/sec) to high (ω = 100 rad/sec) were performed on (**a**) 2:10, (**b**) 2:5, (**c**) 1:10, and (**d**) 1:5 PNP hydrogel formulations. Storage moduli remained above loss moduli at all frequencies tested, indicating solid-like properties of the gels.


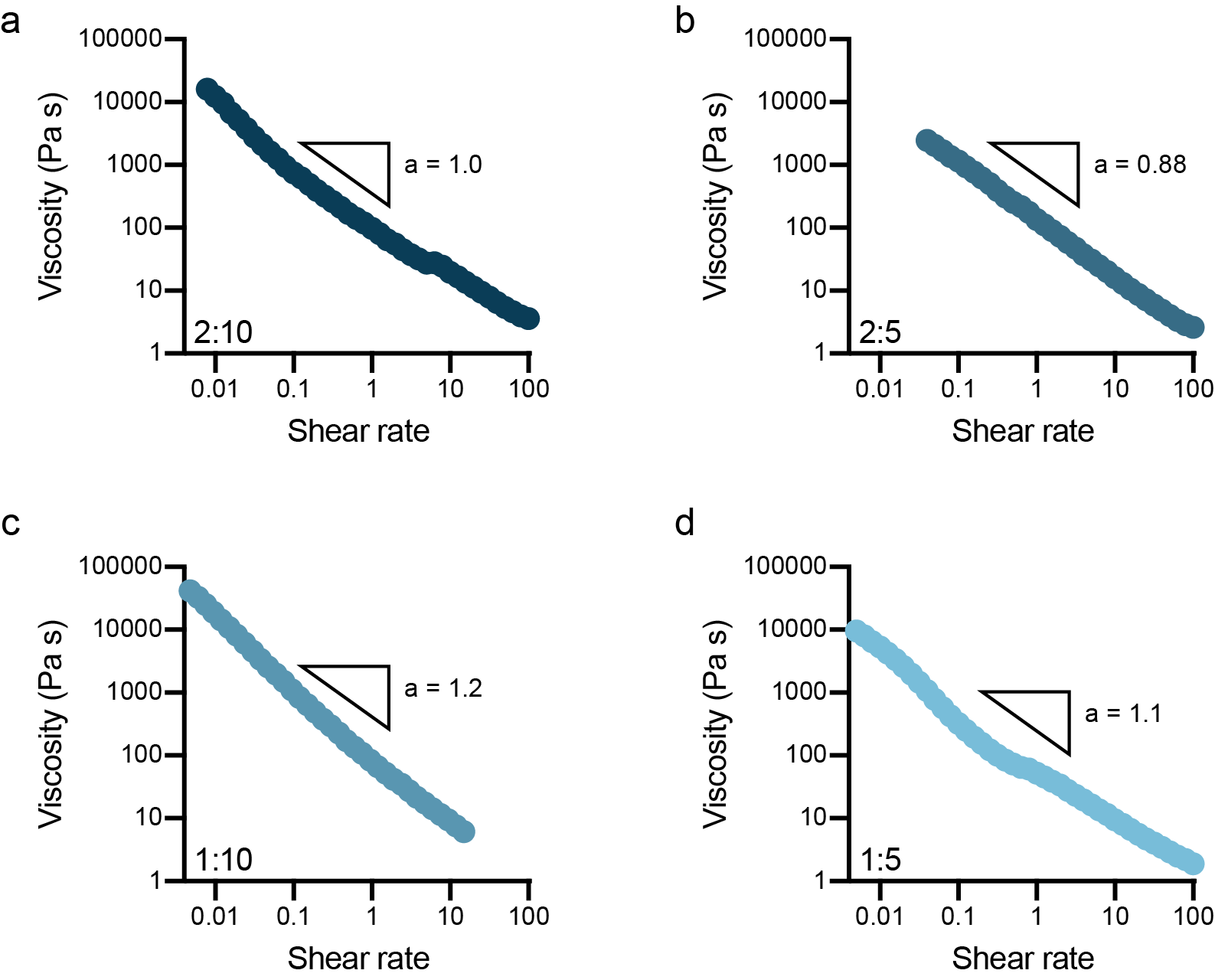


**Figure S3 | Viscosity measurements from shear rate sweeps of each gel formulation.** Shear rate sweeps from high (a, b, d: 100 sec^-1^, c: 15 sec^-1^) to low (<0.05 sec^-1^) were performed on (**a**) 2:10, (**b**) 2:5, (**c**) 1:10, and (**d**) 1:5 PNP hydrogel formulations. The shear thinning index “a” was greater than 1 for all formulations, indicating shear-thinning behavior and injectability.


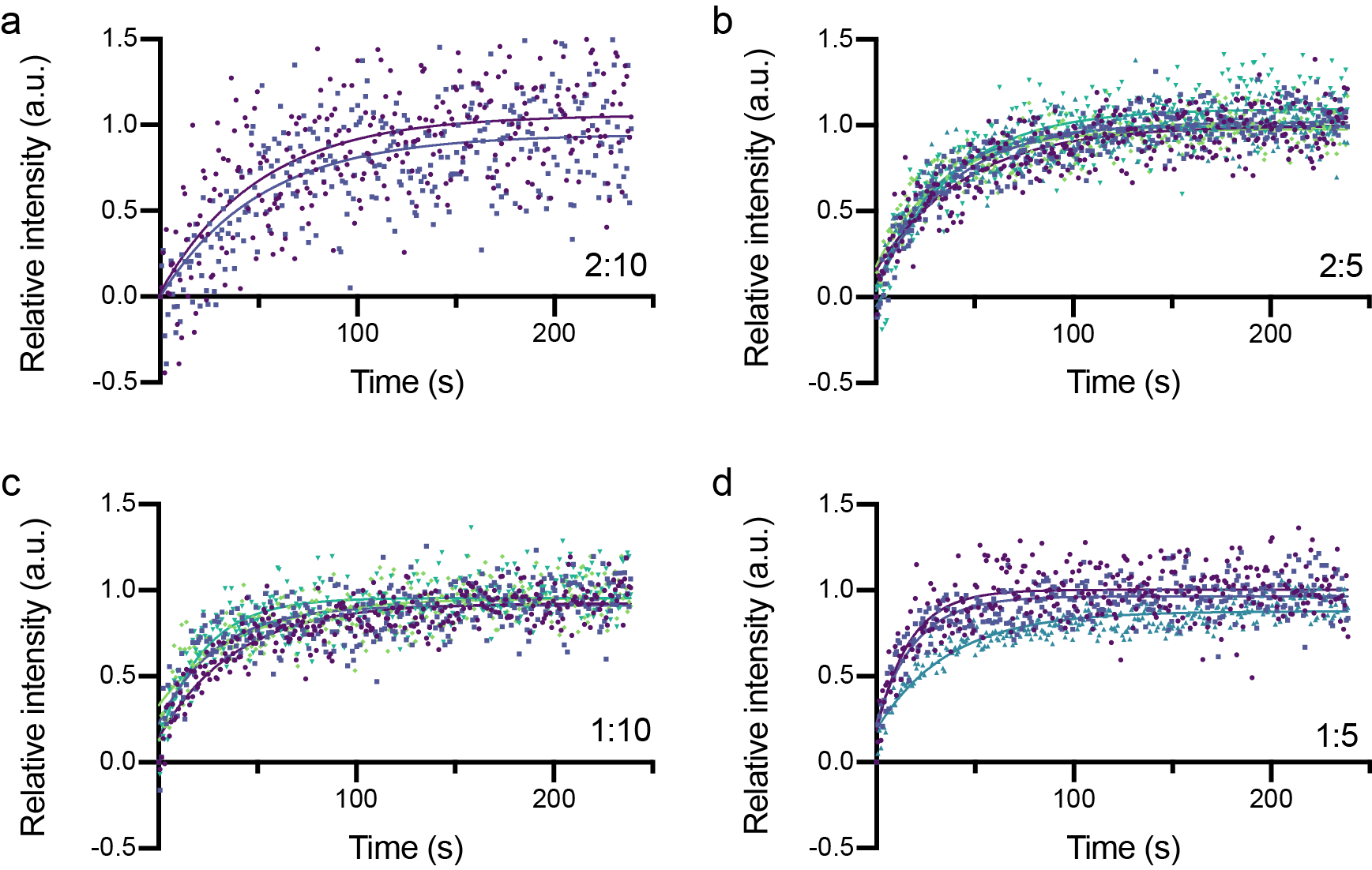
­

**Figure S4 | Fluorescence recovery curves of fluorescently-tagged hemagglutinin in gels.** FRAP measurements were conducted on each gel formulation loaded with HIS-Lite tagged HA (a, n = 2; b,c, n = 5; d, n = 3). Curves were fit to the data and the diffusion coefficient was calculated according Axelrod’s model^1^.

**
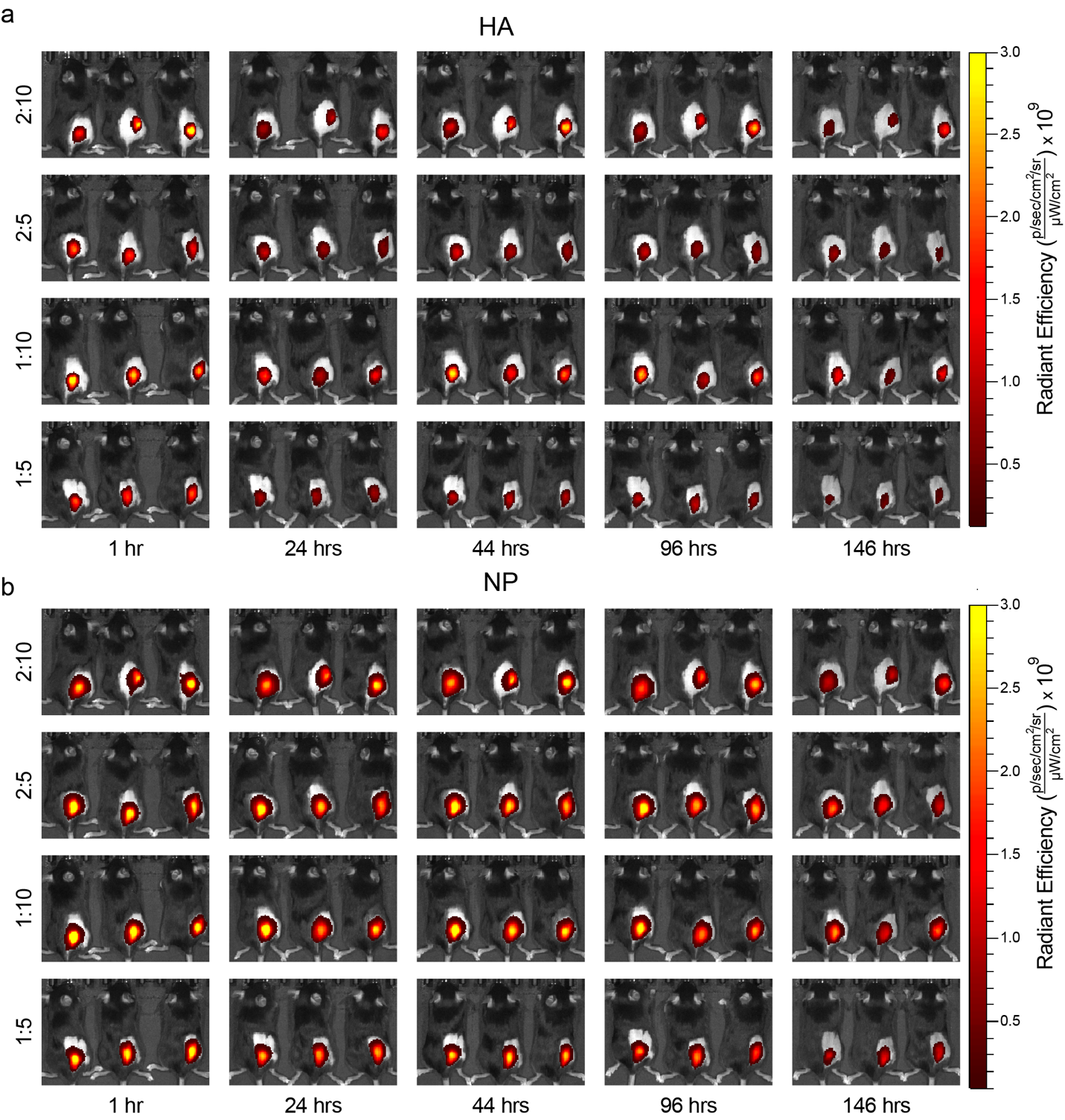
**

**Figure S5 | IVIS images of fluorescently tagged vaccine cargo in mice.** Gels with varied polymer : nanoparticle ratios containing fluorescently tagged HA and NPs were administered in mice (n=3) and imaged at a series of timepoints post injection. The images display that the dissipation of the fluorescently tagged cargo occurs the most quickly in the weakest 1:5 gel.

**Supplementary Tables**

## **Table S1 | Cargo and Polymer Diffusivities** **in 2:10 PNP hydrogel (Measured with FRAP)**

| **Sample** | **Diffusivity (μm^2^/s)** |
| --- | --- |
| NP (n=2) | 1. ± 0.48 |
| HA (n=2) | 1.1 ± 0.18 |
| HPMC-C_12_ (n=2) | 1.4 ± 0.21 |

**References**

1. Axelrod D. et al. Mobility measurement by analysis of fluorescence photobleaching recovery kinetics. Biophys J. 1976;16(9):1055-69.
